# A multi-criteria framework for disease surveillance site selection: case study for *Plasmodium knowlesi* malaria in Indonesia

**DOI:** 10.1098/rsos.230641

**Published:** 2024-01-10

**Authors:** Lucinda E. Harrison, Jennifer A. Flegg, Ruarai Tobin, Inke N. D. Lubis, Rintis Noviyanti, Matthew J. Grigg, Freya M. Shearer, David J. Price

**Affiliations:** ^1^ School of Mathematics and Statistics, The University of Melbourne, Melbourne, Australia; ^2^ Melbourne School of Population and Global Health, The University of Melbourne, Melbourne, Australia; ^3^ Department of Paediatrics, Faculty of Medicine, Universitas Sumatera Utara, Medan, Indonesia; ^4^ Eijkman Institute for Infection and Molecular Biology, Jakarta, Indonesia; ^5^ Menzies School of Health Research and Charles Darwin University, Darwin, Australia; ^6^ University of Melbourne, at the Doherty Institute for Infection and Immunity, Melbourne, Australia

**Keywords:** geospatial modelling, disease surveillance, site selection, *Plasmodium knowlesi* malaria, multi-criteria decision-making

## Abstract

Disease surveillance aims to collect data at different times or locations, to assist public health authorities to respond appropriately. Surveillance of the simian malaria parasite, *Plasmodium knowlesi*, is sparse in some endemic areas and the spatial extent of transmission is uncertain. Zoonotic transmission of *Plasmodium knowlesi* has been demonstrated throughout Southeast Asia and represents a major hurdle to regional malaria elimination efforts. Given an arbitrary spatial prediction of relative disease risk, we develop a flexible framework for surveillance site selection, drawing on principles from multi-criteria decision-making. To demonstrate the utility of our framework, we apply it to the case study of *Plasmodium knowlesi* malaria surveillance site selection in western Indonesia. We demonstrate how statistical predictions of relative disease risk can be quantitatively incorporated into public health decision-making, with specific application to active human surveillance of zoonotic malaria. This approach can be used in other contexts to extend the utility of modelling outputs.

## 1. Introduction

Disease surveillance aims to collect data at different times or locations, to improve our understanding of the spatial and/or temporal distribution of a disease [1–3]. Surveillance data assist public health authorities to distribute control measures, vaccines and medical resources where and when they are needed [2,4]. Disease surveillance is typically limited by budget, time and resources [5–7]. Surveillance activities should therefore be optimized subject to specific stakeholder priorities.

In a spatial context, the targeted allocation of public health resources should incorporate knowledge of the spatial distribution of the disease [2]. There is high uncertainty in many such estimated distributions: sampling bias [8], diagnostic or geographical limitations in an underlying case dataset [9], poor understanding around modes of disease transmission or specific reservoir or vector species [10], and model covariate choice all contribute to uncertainty in a final model estimate. In an ideal setting, surveillance would have complete coverage over a study area, although this is rarely an option—instead, we must consider the best way to distribute surveillance resources, given uncertainty [1,11]. We should consider what exactly ‘best’ means to our specific surveillance context. In other words, we should form a sampling objective.

All disease surveillance should be carried out with a clearly defined set of objectives. There are many possible goals of surveillance: for example, we may aim to identify infections so that appropriate treatment can be administered, or to improve our overall understanding of a disease’s spatial distribution [11] or transmission dynamics [12], to allow monitoring and/or to predict future changes. The immediate priorities of surveillance may complement or compete with longer-term motivations. Priorities also depend on the disease context—the aims of surveillance in a disease outbreak setting [5] may be contradictory to those of endemic disease surveillance [11]. Given one or more objectives, there may be constraints on surveillance, for example, relating to cost and workforce [13]. The range of stakeholders with an invested interest in surveillance may be broad, each with their own specific priorities.

Existing methods of optimized site selection in ecology and epidemiology have often been constructed around specific sampling objectives [7,13–15]. Ferrier *et al.* [14] and Grist *et al.* [13] describe site selection workflows involving spatial distribution models related to turnover in species diversity and *Plasmodium falciparum* anti-malarial drug resistance, respectively. Longbottom *et al.* [7] develop a site selection method which includes an estimated spatial distribution of disease as a constraint, rather than as part of a quantitative objective, with the model output used to define the area where the population is at risk of disease. Each of these methods assumes a clear, specific objective. However, in many possible use cases—particularly when multiple stakeholders are involved—a single sampling objective may not be clear.

Multi-criteria decision analysis (MCDA) is an established approach that formalizes an objective into an explicit function of decision criteria [16]. In a spatial setting, decision criteria can be represented by data layers. Criteria which may be relevant to human disease surveillance include the estimated disease distribution, site accessibility or human population density [13]. These criteria may be iterated as decision-makers receive new information or change their priorities. MCDA combines multiple criteria into a single decision surface: optimization corresponds to selecting the solution with the single highest objective value. The creation of a decision surface before optimization means that the full consequences of trade-offs made may not be fully understood by the decision-maker [17]. The method is therefore sensitive to individual decisions without the implications of each being clear to the decision-maker.

There are several examples of frameworks which incorporate spatial predictions of disease distributions into public health decision-making (e.g. [7,13]), although these consider single objectives for specific decision problems. Flexibility to consider multiple dynamic and conflicting decision-maker preferences is a key advantage of a multi-criteria approach. In this paper, we develop a novel decision-making framework to quantify disease surveillance objectives and select sites that optimize those objectives. This framework quantitatively includes an estimated geospatial disease distribution into the decision-making process. We demonstrate the framework’s utility by applying it to site selection for human surveillance of *Plasmodium knowlesi* malaria in western Indonesia.

This paper is structured as follows: we first describe methods of site selection, drawing upon MCDA. We then introduce the case study of *Plasmodium knowlesi* malaria surveillance (§2.3) and demonstrate an application of the methods (§3). Finally, we discuss the strengths and limitations of the methods described in the context of the case study and the broader context of surveillance site selection problems (§4). The reader can reproduce our results using our RShiny application (https://lucyharrison.shinyapps.io/pk_multicrit_shiny/). Code and data are made available at https://github.com/lu-harr/pk_multicriteria_framework.

## 2. Methods

### 2.1. Objective and constraints

The method outlined here is inspired by weighted overlay analysis and spatial MCDA [16,18]. We define a set of decision alternatives, x, that we wish to sample from. In our case study, each pixel in the study area is a potential surveillance site, so that the set of potential sites in x can be arranged in a grid. Decision criteria are variables that are relevant to site selection: if x is a grid, each decision criterion is a data layer. For example, if some minimum number of resident humans are required at a candidate surveillance site, human population density is a relevant decision criterion layer. An objective surface can be formed by applying overlay operations to decision criteria, for example, using a weighted sum or weighted product [16,19,20]. The ultimate objective surface assigns a value to each pixel in the study area based on its utility to sampling, i.e. the objective is maximized. We form our objective surface, F(x) from the predictions of a statistical model of disease risk

2.1 $$F(x_{i})=f\;(\mu (x_{i}),\sigma (x_{i}),z_{i}^{1},\ldots ,z_{i}^{n}),$$

where *F*(*x*_*i*_) is the objective value at location *x*_*i*_, *μ*(*x*_*i*_) and *σ*(*x*_*i*_) are the model prediction mean and standard deviation of the pixel *x*_*i*_ in the study area, and $\left\{z_{i}^{1},\ldots ,z_{i}^{n}\right\}$ are further priorities to be included in the objective surface. In the case study (§2.3), we use the relative risk of disease as estimated, with uncertainty, from a bootstrapped boosted regression tree described by Shearer *et al.* [9] (where *μ*(*x*_*i*_) and *σ*(*x*_*i*_) are the mean and standard deviation of the bootstrapped model predictions at pixel *x*_*i*_; see electronic supplementary material, §S2). Standard deviation, *σ*(*x*_*i*_), is used rather than variance to ensure the variability does not dominate the mean and instead scales with the number of bootstraps. However, any geospatial statistical model, or measure of the spatial distribution of a disease, can be substituted in place.

#### 2.1.1. Site catchment: characterizing decision alternatives

**Figure 1.**
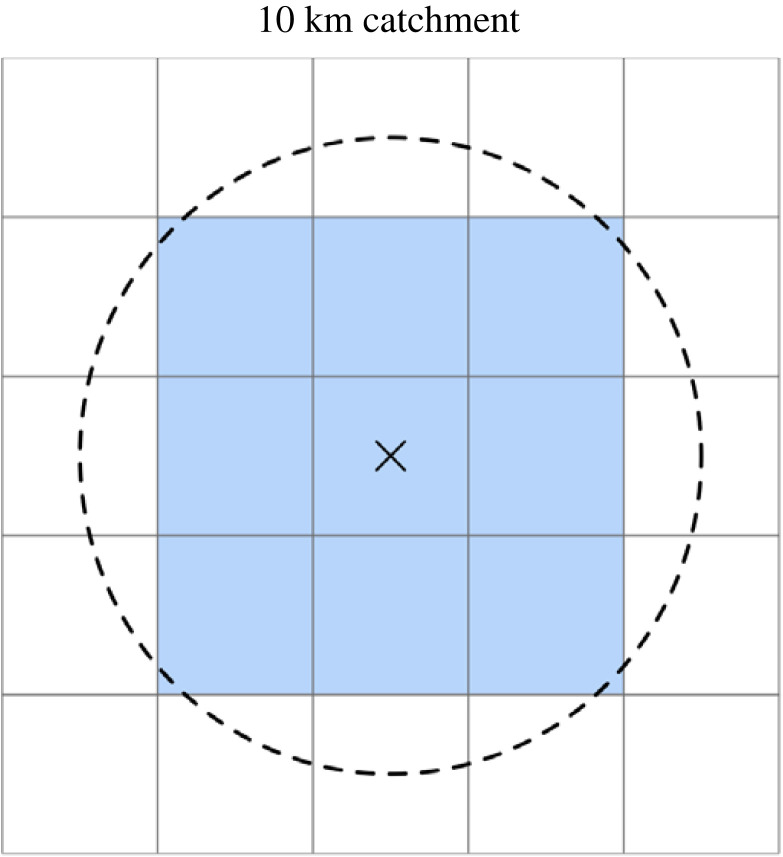
A 10 km catchment for a pixel in a 5 × 5 km^2^ grid. The radius of the catchment is 10 km from the centroid of the pixel (black cross). Other pixels are included into the catchment (blue) if the majority of their area lies inside the circular boundary.

We consider the selection of sites from a grid of decision alternatives. The objective value of each alternative is independent of the value of proximal sites, excluding spatial autocorrelation in objective or constraint surfaces. However, the objective value of proximal sites is likely to be relevant to decision-making. For example, it is possible that a human case recorded in a particular pixel was exposed to the disease in a different pixel, and it is more likely that the case was exposed in an adjacent pixel than a distant one. We characterize a site ‘catchment’: the area around a site that is relevant to a site’s objective value. For example, we could include all pixels within some radius of the selected pixel into its catchment (figure 1). The covariate value of all pixels within a site’s catchment can then be summarized to create an argument of the objective function (equation (2.1)). In §3, the objective value of a pixel given a 10 km catchment is the mean of the initial objective surface at all pixels in the catchment. The use of a hard buffer is a simple way to link the objective values of proximal sites but is only one example of several possible approaches. Catchment definition may relate to the range of a host or vector species and need not be circular—for example, it may factor in a measure of host movement or accessibility.

#### 2.1.2. Uncertainty- and precision-weighted objectives

The selected surveillance objective, a function of modelling outputs as in equation (2.1), should align with the specific aims of a surveillance programme. In this paper, we explore two example objectives

2.2 $$F_{1}(x_{i})=\mu (x_{i})\times \sigma (x_{i})$$

and

2.3 $$F_{2}(x_{i})=\mu (x_{i})\times \frac{1}{\sigma (x_{i})},$$

designated the ‘uncertainty-weighted’ and ‘precision-weighted’ objectives, respectively. These objectives focus only on model prediction mean and standard deviation, with no further $z_{i}^{j}$ (equation (2.1)). The uncertainty-weighted objective (equation (2.2)) assigns the highest value of *F*_1_( · ) to sites with high prediction mean and high uncertainty. In the context of disease surveillance, this prioritizes sites where modelled transmission risk suggests a higher likelihood of identifying an infection, but where the model is also uncertain. This objective aims to select sites where we will ‘learn’ the most about disease presence, or where inclusion in future model fitting will most reduce model uncertainty. For example, in a disease introduction setting, there may be areas where it is known that the disease is present or absent with high confidence. Stakeholders may then prioritize surveillance in areas where uncertainty is high, but modelling (or other approaches to risk assessment) suggests the disease could be present.

The precision-weighted objective (equation (2.3)) assigns the highest value of *F*_2_( · ) to sites with high prediction mean and low uncertainty (equivalent to the ‘signal-to-noise ratio’ [21,22] or the inverse of the ‘coefficient of variation’ [23]). This objective targets sites where the model is most confident that an infection will be identified. For example, in a setting where resources for treatment are limited, surveillance may be prioritized in areas where positive detections of the pathogen are likely.

#### 2.1.3. Constraining the objective

As well as criterion layers directly related to the distribution of disease, criterion layers capturing other surveillance aims may be included in the objective function as further factors, $z^{j}$ (equation (2.1)). There are often practical limitations on surveillance; examples of useful factors include a layer to delineate areas occupied by the population at risk of the disease [7,9], or a layer of site accessibility or human population density, to down-weight areas in the broader sampling region where human surveillance is less feasible [13]. There may also be environmental criteria for site selection, such as proximity to a watercourse, primary forest or croplands. Grist *et al.* [13] consider ‘thresholds’ of model uncertainty, a factor already included in their objective, to prioritize sites where uncertainty is above a certain level. Where the aim is to separate the full set of sites in the study area into those that are feasible for surveillance and those that are infeasible, a criterion layer may be considered as a constraint.

Each constraint can be introduced into the objective via the application of a *constraint function* (or utility function [16]), *C*( · ), to a criterion layer, $z^{j}$. The criterion layer is scaled according to the decision-maker’s preference. The resulting *constraint layers* then scale the objective surface (equation (2.1)) as a further *m* factors

2.4 $$F(x_{i})=F_{\alpha }(x_{i})\times C_{1}(z_{i}^{1})\times C_{2}(z_{i}^{2})\times \cdots \times C_{m}(z_{i}^{m}),$$

where $F_{\alpha }(\cdot )$ corresponds to the uncertainty-weighted objective, *F*_1_( · ), or the precision-weighted objective, *F*_2_( · ).

Malczewski [16] distinguishes two classes of decision constraint: ‘Boolean’ and ‘target’ (electronic supplementary material, figure S1). Under a Boolean constraint, sites are feasible or infeasible for selection based on their corresponding value in a criterion layer being less than (or greater than) a threshold (i.e. a one-way exclusion). Under a targeted constraint, sites are feasible or infeasible based on their position within an acceptable range (i.e. a two-way exclusion with two threshold values). An example constraint function for a Boolean constraint may be

2.5 $$C(z_{i})=\left\{ \begin{array}{cc} 1, & z_{i}\geq k\\ 0, & z_{i}<k \end{array}\right..$$

Similarly, for a targeted constraint, the value of a constraint function is determined by the presence of $z_{i}^{j}$ within a target interval:

2.6 $$C(z_{i})=\left\{ \begin{array}{cc} 1, & z_{i}\in [k_{\mathrm{low}},k_{\mathrm{high}}]\\ 0, & z_{i}\notin [k_{\mathrm{low}},k_{\mathrm{high}}] \end{array}\right..$$

Alternatively, if the decision-maker does not wish to entirely exclude all sites via a threshold, to avoid large differences in objective value from small differences in criterion value, a ‘softer’ constraint function can be applied (electronic supplementary material, figure S1).

A relevant constraint to the selection of *P. knowlesi* malaria surveillance sites is that of site accessibility. An accessibility surface is derived from the friction surface of global travel time published by Weiss *et al.* [24] (electronic supplementary material, §S3). Another relevant constraint to the case study is that of proportional forest cover. Both the wildlife reservoir species and mosquito vector species are historically forest-dwelling [25]: stakeholders may seek to exclude sites which do not contain forest or forest-edge habitats for reasons of environmental suitability. An aggregated forest surface is derived from the Intact Forest Landscapes dataset published by Potapov *et al.* [26] (electronic supplementary material, §S3). In this application, both site accessibility and forest cover are examples of Boolean constraints, as sites below some threshold of these features are those that are unsuitable for surveillance (electronic supplementary material, figure S3).

### 2.2. Selection of sites

Having created a final, constrained, objective surface, we now consider the process of site selection. The most straightforward method of site selection given an objective surface is to simply select the sites with highest objective value, as demonstrated by Grist *et al.* [13]. Under both the uncertainty-weighted and precision-weighted objectives described in equations (2.2) and (2.3) (and the objective suggested by Grist *et al.* [13]), the objective value of successively selected sites is independent of the set of sites previously selected. However, there are some practical constraints for which the objective value at a site should depend on other sites selected. For example, we could constrain the distance between sites to be greater than a specific threshold, to spread sites out in the study area. A site’s objective value would then depend on whether another site is selected within the threshold distance. For example, if the sites with the first- and second-highest objective values in the study area are within the threshold distance of each other, the site with the second-highest objective value is excluded from selection. In this case, the optimal solution cannot be found by selecting sites one at a time.

When the number of sites to be selected and the sampling frame are prohibitively large, approximate methods of optimal site selection are required. A greedy site selection algorithm, which selects sites one at a time, recalculating the objective after each selection, is outlined in algorithm 1 [27]. Another approximate solution is to sample only a portion of the set of possible site combinations and compare their aggregated objective values, as is demonstrated by Longbottom *et al.* [7].

In this work, there is no feedback of surveillance data to decision criteria (i.e. modelling outputs) between the selection of sites—the use of new information from each successive site would make optimization *adaptive* [28]. Instead, the recalculation of the objective, given the set of selected sites, involves changing interactions between decision criteria and constraints. Algorithm 1 could be applied in an adaptive setting: modelling steps would be repeated as data is recorded at sites and the values of decision criteria would change for each new site to be selected.


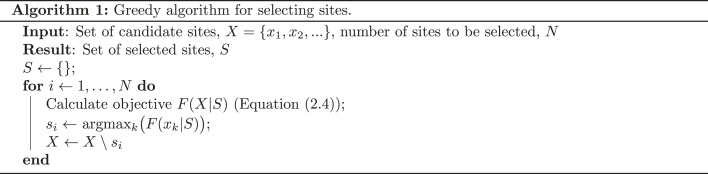


### 2.3. Case study: human surveillance of *Plasmodium knowlesi* malaria in Indonesia

**Figure 2.**
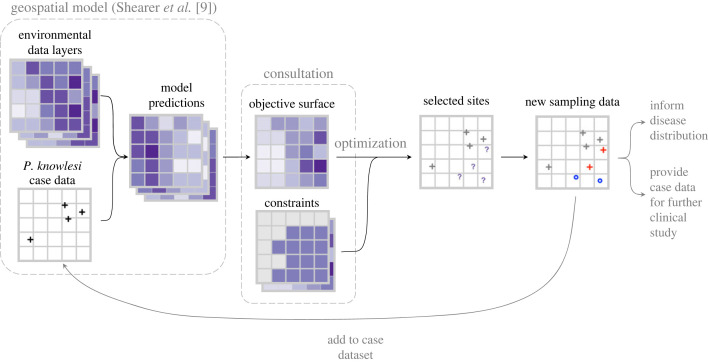
Framework for *Plasmodium knowlesi* malaria case study. Existing case data and gridded environmental data are inputs to a model of relative *P. knowlesi* malaria risk [9]. The objective surface, a function of model predictions, is combined with a set of constraints through stakeholder consultation to select sites for surveillance (purple question marks). During surveillance, *P. knowlesi* malaria is either recorded at a site (red cross), or is not (blue circle). Surveillance data can be added to the case dataset to update the geospatial model.

In this paper, we consider the application of a generalizable framework for site selection to human surveillance of *Plasmodium knowlesi* malaria (figure 2). Zoonotic *P. knowlesi* is now the most common cause of human malaria in Malaysia [29]. The parasite is increasingly reported elsewhere in the Southeast Asia region, including in western Indonesia [12,30]. Unlike other human *Plasmodium* species, *P. knowlesi* is transmitted to humans via mosquitoes from several macaque species that are endemic to Southeast Asia: *Macaca nemestrina*, *M. fascicularis* and *M. leonina* [31]. Anopheline mosquitoes of the Leucosphyrus group act as vectors [31]. The parasite is commonly misidentified by microscopy as either *P. malariae* or *P. falciparum* [32]; a more resource-intensive molecular assay is required to accurately identify *P. knowlesi*. Testing equipment is expensive and is not available in many endemic areas [12]. Together with a large suspected proportion of unreported or asymptomatic cases [11], this has resulted in a substantial degree of spatial bias in the existing *P. knowlesi* malaria case dataset. The spatial extent of human infection, particularly outside of Malaysia, is not well understood [9,25]. It is therefore critical to prioritize the spatial allocation of febrile illness sampling effort, with samples generally sent to a central laboratory with appropriate testing capacity.

Deforestation has been identified to affect the distributions and behaviour of both macaque host and mosquito vector species, by fragmenting or destroying macaque habitat and providing breeding sites for vectors [25,33]. The replacement of primary forest with oil palm plantation, and other agricultural expansion, creates an environment of increased risk: macaques, vectors and humans are brought into close proximity [25,34]. Men of working age in forest or farm-based occupations have been identified to be at increased risk of *P. knowlesi* malaria infection [34,35].

Previous *P. knowlesi* malaria human surveillance efforts in Indonesia have been organized with only limited infection records [9,12]. As a result, study sites have been selected based on observations of other variables related to transmission, including the presence of alternative macaque host species, data of broader human malaria endemicity [12] and appraisals of logistical suitability.

There are several existing spatial distribution models of *P. knowlesi* malaria risk [9,36,37]. While other models predict *P. knowlesi* malaria risk on a district or state level, a key distinction of the model described by Shearer *et al.* [9] is a map with a resolution of approximately 5 × 5 km^2^ of relative predicted risk throughout the Southeast Asia region [9] (electronic supplementary material, §S2). Given the high degree of uncertainty in the known spatial distribution of *P. knowlesi* malaria, we use this map to support the selection of sites for planned surveillance of *P. knowlesi* malaria in humans in consultation with project stakeholders (see figure 2 for framework overview). Sites will be selected concurrently—surveillance data from the highest ranked selected sites will not be available to shape decisions on later sites. However, the framework presented is flexible to changing stakeholder objectives and we expect that risk models and objectives will be updated, subject to case data recorded at selected study sites, and as disease surveillance continues into the future (as shown in figure 2).

A description of the model published by Shearer *et al.* [9] is included in electronic supplementary material, §S2. Briefly, the model is a boosted regression tree trained on a dataset of geolocated *P. knowlesi* malaria cases in Malaysia, Brunei and Singapore. Model covariates include surfaces of land cover, human presence and macaque host species and mosquito vector species distribution [31]. The model is fit to 500 bootstrapped versions of the initial training dataset to produce 500 prediction surfaces at a resolution of approximately 5 × 5 km^2^. These prediction surfaces are aggregated for use in site selection (i.e. to evaluate the mean and variance in predicted relative risk). All training and covariate data referred to in this paper are identical to those discussed in Shearer *et al.* [9]. Minor adjustments to the model code (https://github.com/fshearer/pk_parasite) were made in response to software deprecation. Further detail is provided in electronic supplementary material, §S2.

#### 2.3.1. Communication of method

**Figure 3.**
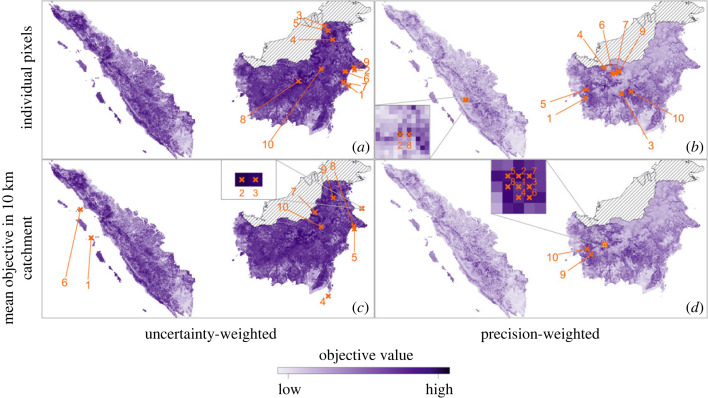
The uncertainty-weighted ((*a*,*c*); equation (2.2)) and precision-weighted ((*b*,*d*); equation (2.3)) objective surfaces, in the absence of constraints. The 10 individual sites with highest value under each objective are also plotted, for individual pixels (*a*,*b*) or 10 km radius catchments (*c*,*d*). Sites are labelled from 1 to 10 in order of decreasing objective value.

**Figure 4.**
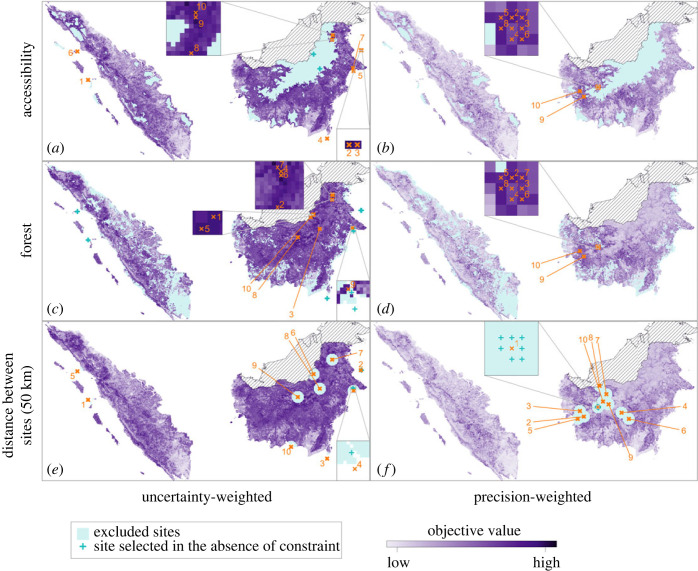
Uncertainty-weighted (*a*,*c*,*e*) and precision-weighted (*b*,*d*,*f*) objective surfaces, with the following constraints applied: (*a*,*b*) accessibility (electronic supplementary material, figure S3A); (*c*,*d*) forest (electronic supplementary material, figure S3B); (*e*,*f*) distance between sites, applied using greedy site selection (algorithm 1), so that areas within 50 km of each successively selected site are excluded. Excluded areas are shaded in blue. Sites selected given the constraint (orange), and sites selected in the absence of constraint (blue; figure 3*a*,*b*) are included.

To aid in the communication of the quantitative method for site selection and to increase its utility, an application was created using R’s Shiny package [38] (https://lucyharrison.shinyapps.io/pk_multicrit_shiny/). This application allows project stakeholders to interact with the objective surface and constraints, to better understand the trade-offs of individual decisions, including the choice of constraint surfaces and thresholds. The priority of this work is to supply stakeholders with quantitative support in the planning stage of a study—the communication of the recommendations of the site selection framework is therefore a key outcome. Panels from figures 3 and 4 can be generated in the application. Code for the application is available at https://github.com/lu-harr/pk_multicriteria_framework.

## 3. Results

### 3.1. Unconstrained site selection

We first explore the application of both uncertainty-weighted (equation (2.2)) and precision-weighted (equation (2.3)) sampling objectives to the surveillance of *P. knowlesi* malaria in the regions of Sumatra and Kalimantan in western Indonesia (figure 3*a*,*b*), given the predictions of the existing geospatial model of *P. knowlesi* malaria risk [9]. The 10 pixels with the highest objective value are labelled for each objective surface in figure 3*a*,*b*.

Under the uncertainty-weighted objective, the first 10 sites selected are all in northeastern Kalimantan (figure 3*a*). The uncertainty surface is relatively high in this region, particularly in areas bordering Malaysian Borneo (electronic supplementary material, figure S2B), where forest is largely intact (electronic supplementary material, figure S3B). By contrast, many of the first 10 sites selected under the precision-weighted objective are in western Kalimantan (figure 3*b*). The mean surface contains generally higher values in this area and uncertainty is generally lower (electronic supplementary material, figure S2).

Both the uncertainty- and precision-weighted objectives demonstrated in figure 3 rely on a balance between model prediction mean and standard deviation. Electronic supplementary material, figure S4A demonstrates the effects of mean and standard deviation on the uncertainty-weighted objective: a site with high mean and low standard deviation may have equal objective value to a site with low mean and high standard deviation. Therefore, equal priority will be assigned to a site where the model is uncertain but the underlying relative risk may be high, and a site where the model is confident that the underlying relative risk is high. In any case, these sites have lower objective value than sites with both high mean and high uncertainty—in the case study, we select only a small fraction of the full set of sites, so sites which optimize both decision criteria are selected. Electronic supplementary material, figure S4B demonstrates a similar trade-off under the precision-weighted objective: as the standard deviation decreases towards zero, its influence on objective value increases by orders of magnitude, to the extent that sites with low mean and extremely low standard deviation may be ranked above sites with high mean and only moderately low standard deviation. High mean predicted disease risk may implicitly be a more important criterion than low uncertainty, although this preference is not explicitly included in the objective function in equation (2.3). This objective is less suited to applications where uncertainty in predicted risk is relatively low. Ultimately, the application of the precision-weighted objective to *P. knowlesi* malaria surveillance site selection is motivated by the high degree of uncertainty in model predictions of *P. knowlesi* malaria relative risk. The relationships between constituent factors of the objective should be reviewed to ensure that the situations it aims to identify are appropriately captured.

#### 3.1.1. Unconstrained site selection given site catchments

The selection of sites under the uncertainty- and precision-weighted objectives given 10 km radius catchments (figure 1) is demonstrated in figure 3*c*,*d*. The objective value used to select sites is the mean of the objective values of all sites in a given catchment: this increases the spatial autocorrelation, or ‘blurring’, in the objective surface. Under the uncertainty-weighted objective (figure 3*c*), this ‘blur’ is reflected in the large number of island sites selected. Islands generally have high values in the uncertainty surface, the catchments of island pixels are generally smaller (e.g. sites 2 and 3 in figure 3*c* have catchments of only two pixels each) and the shape of the uncertainty-weighted objective distribution is such that there is a low number of high-objective pixels (electronic supplementary material, figure S4). Under the precision-weighted objective, the ‘blur’ is visible in that the eight sites with the highest objective values are direct neighbours, none of which are selected when objective value does not depend on decision criteria at adjacent sites (figure 3*b*).

### 3.2. Constrained site selection

Figure 4 shows the application of three distinct constraints to the objectives explored in figure 3. An example of a Boolean constraint on site accessibility (equation (2.5)) is applied to the uncertainty- and precision-weighted objectives in figure 4*a*,*b*. The 20% of sites with the lowest site accessibility are excluded from selection (see electronic supplementary material, §S3 and figure S3A). The area most excluded by this constraint is north-central Kalimantan—this region contains large areas of intact forest. Sites excluded from selection by this constraint are likely to be more costly to access than others in the study area, so may be less feasible for surveillance. The change to the set of sites selected when the site accessibility constraint is imposed are reflected in figure 4*a*,*b*. All selected sites that are excluded under the uncertainty-weighted objective are in north-central Kalimantan, the area most penalized by the urban accessibility constraint.

A second Boolean constraint, on proportional forest cover at a site, is applied in figure 4*c*,*d*. Like the accessibility constraint, the 20% of sites with lowest forest cover are excluded from selection. Southern and central Sumatra are the areas most affected, as well as coastal areas in Kalimantan. Under the uncertainty-weighted objective, four of the 10 best sites (figure 3*a*) are excluded when forest is constrained; these are all in coastal east Kalimantan (figure 4*c*). The set of sites selected under the precision-weighted objective (figure 3*b*) does not change when the forest constraint is imposed (figure 4*d*).

A final constraint, on minimum distance between selected sites, is also applied in figure 4*e* and *f*: a 50 km ‘exclusion zone’ is applied around each selected site (i.e. sites within this zone are removed from the set of candidate sites as each new site is selected). This constraint is relevant to policy as surveillance at sites which are proximal limits the coverage of a study. More generally, sampling at two proximal sites may lead to interference in results. When the minimum distance constraint is applied, the objective value of each site is dependent on the locations of other selected sites, in contrast to the site accessibility and forest cover constraints described in §2.1.3. As the number of combinations of possible site selections is prohibitive, we apply a greedy search algorithm (algorithm 1) to find a constrained set of sites for both the uncertainty- and precision-weighted objectives in figure 4*e*,*f*. Two sites from the unconstrained sets are excluded under both objectives. To demonstrate the application of constraints to site catchments, figure 4 is replicated for 10 km radius catchments in electronic supplementary material, figure S5.

## 4. Discussion

In this paper, we outline a framework that quantitatively incorporates the predictions of a geospatial statistical model of relative disease risk into public health decision support. We demonstrate the utility of this framework for site selection for surveillance of the zoonotic malaria parasite, *P. knowlesi*, in western Indonesia. The framework, which is generalizable to other pathogens, models, objectives and geospatial decision problems, is the primary novel contribution of this work. Its communicability to project stakeholders and its ability to be subsequently developed, given feedback, is a key strength of our approach.

The MCDA-inspired method described involves decision-making (i.e. the selection of objective factors and constraints) before optimization [17]. This simplifies the optimization process, as only one objective function is considered during site selection, although the set of selected sites is highly sensitive to the preferences of the decision-maker. This is a sensible attribute of the framework, as we aim to provide a quantitative method to support site selection under a specific set of surveillance goals. However, it is highly likely that surveillance goals will change over the lifetime of a surveillance programme. It is for this reason that we emphasize the flexible nature of the framework (figure 2) and the intention that surveillance data be eventually fed back into modelling and consultation steps as a programme evolves. The use of interactive applications and the presentation of alternative choices allow the decision-maker to better understand the trade-offs involved in each individual decision.

A group of public health colleagues has provided feedback and further isolated the specific sampling scenario and aims of planned *P. knowlesi* malaria surveillance. Our colleagues expressed a preference for the precision-weighted objective surface—this objective prioritizes sites where a case is likely to be detected. This objective is selected due to the high degree of uncertainty in the current spatial disease distribution and the limited number of sites available for selection. While formal validation of the framework is not currently planned, the data produced by surveillance activities will feed back into the case dataset that informs the geospatial distribution model, which will shape future decision-making for disease surveillance. Indeed, this iterative approach, in consultation with public health decision-makers, is an important aspect of ongoing surveillance activities (as outlined in figure 2). However, the time frame over which data are collated and able to be incorporated into the model, such that updated surveillance designs can be evaluated, is important (within a transmission season, between seasons, after multiple seasons etc.). While beyond the scope of this study, the approach described here for a single design evaluation could simply be extended to consider multiple iterations as new data are made available, as public health priorities shift, and in the presence of new constraints.

The method described in this work represents an initial step in the use of quantitative methods for *P. knowlesi* malaria surveillance site selection in western Indonesia. Unlike existing site selection workflows that incorporate the outputs of geospatial models [13,14], the framework presented here involves a sampling objective that can be tailored to specific project aims. The motivation for this work is to allow geospatial models of disease risk to be quantitatively included into public health decision-making.

The methodology we present here is generalizable to other decision problems. Rather than being motivated by a specific objective, this framework includes the selection of an objective and constraints, and the quantitative incorporation of geospatial information relevant to the selected objective into the site selection process. In public health and epidemiological research, quantitative analysis is often employed after data has been collected. Our method is intended to drive study planning, to reduce sampling bias and to improve the quality of the data that serves as input to downstream analysis. The Shiny app presented with this publication translates the framework into a format that allows decision-makers to interact with the trade-offs involved in their decisions. The framework extends the utility of geospatial disease models to directly include their predictions and associated uncertainty in operational decision-making.

## Author summary

*Plasmodium knowlesi* malaria is transmitted to humans from monkeys via mosquitoes across Southeast Asia. We can predict where the risk of disease is high using environmental data related to monkey and mosquito habitat. We prioritize areas to look for the disease, based on these predictions and accounting for uncertainty.

## Data Availability

Data and relevant code for this research work are stored in GitHub: https://github.com/lu-harr/pk_multicriteria_framework and have been archived within the Zenodo repository: https://doi.org/10.5281/zenodo.10247166 [39]. Supplementary material is available online [40].

## References

[1] Diggle P, Ribeiro P. 2007 Geostatistical design. In *Model-based geostatistics*, pp. 199–212. Berlin, Germany: Springer (10.1007/978-0-387-48536-2_8).

[2] Downs J, Vaziri M, Deskins G, Kellner W, Miley K, Unnasch T. 2020 Optimizing arbovirus surveillance using risk mapping and coverage modelling. Ann. GIS **26**, 13-23. (10.1080/19475683.2019.1688391)

[3] Hay S, George D, Moyes C, Brownstein J. 2013 Big data opportunities for global infectious disease surveillance. PLoS Med. **10**, e1001413. (10.1371/journal.pmed.1001413) 23565065 PMC3614504

[4] Herdiana H et al. 2016 Malaria risk factor assessment using active and passive surveillance data from Aceh Besar, Indonesia, a low endemic, malaria elimination setting with *Plasmodium knowlesi*, *Plasmodium vivax*, and *Plasmodium falciparum*. Malar. J. **15**, 468. (10.1186/s12936-016-1523-z) 27619000 PMC5020529

[5] Ritchie S et al. 2007 Operational trials of remote mosquito trap systems for Japanese encephalitis virus surveillance in the Torres Strait, Australia. Vector-Borne Zoonotic Dis. **7**, 497-506. (10.1089/vbz.2006.0643) 18021024

[6] Chevalier V, Lecollinet S, Durand B. 2011 West Nile virus in Europe: a comparison of surveillance system designs in a changing epidemiological context. Vector-Borne Zoonotic Dis. **11**, 1085-1091. (10.1089/vbz.2010.0234) 21548765

[7] Longbottom J, Wamboga C, Bessell P, Torr S, Stanton M. 2021 Optimising passive surveillance of a neglected tropical disease in the era of elimination: a modelling study. PLoS Negl. Trop. Dis. **15**, e0008599. (10.1371/journal.pntd.0008599) 33651803 PMC7954327

[8] Phillips S, Dudík M, Elith J, Graham C, Lehmann A, Leathwick J, Ferrier S. 2009 Sample selection bias and presence-only distribution models: implications for background and pseudo-absence data. Ecol. Appl. **19**, 181-197. (10.1890/07-2153.1) 19323182

[9] Shearer F et al. 2016 Estimating geographical variation in the risk of zoonotic *Plasmodium knowlesi* infection in countries eliminating malaria. PLoS Negl. Trop. Dis. **10**, e0004915. (10.1371/journal.pntd.0004915) 27494405 PMC4975412

[10] Moyes C et al. 2014 Defining the geographical range of the *Plasmodium knowlesi* reservoir. PLoS Negl. Trop. Dis. **8**, e2780. (10.1371/journal.pntd.0002780) 24676231 PMC3967999

[11] Fornace K, Reyes R, Macalinao M, Bareng A, Luchavez J, Hafalla J, Espino F, Drakeley C. 2020 Disentangling fine-scale effects of environment on malaria detection and infection to design risk-based disease surveillance systems in changing landscapes. *medRxiv*. (10.1101/2020.04.15.20065656) PMC8866131 34883232

[12] Lubis I, Wijaya H, Lubis M, Lubis C, Divis P, Beshir K, Sutherland C. 2017 Contribution of *Plasmodium knowlesi* to multispecies human malaria infections in North Sumatera, Indonesia. J. Infect. Dis. **215**, 1148-1155. (10.1093/infdis/jix091) 28201638 PMC5426374

[13] Grist E et al. 2016 Optimal health and disease management using spatial uncertainty: a geographic characterization of emergent artemisinin-resistant *Plasmodium falciparum* distributions in Southeast Asia. Int. J. Health Geogr. **15**, 37. (10.1186/s12942-016-0064-6) 27776514 PMC5078981

[14] Ferrier S, Manion G, Elith J, Richardson K. 2007 Using generalized dissimilarity modelling to analyse and predict patterns of beta diversity in regional biodiversity assessment. Divers. Distrib. **13**, 252-264. (10.1186/s12942-016-0064-6)

[15] Reich B, Pacifici K, Stallings J. 2018 Integrating auxiliary data in optimal spatial design for species distribution modelling. Methods Ecol. Evol. **9**, 1626-1637. (10.1111/2041-210X.13002)

[16] Malczewski J. 1999 GIS and multicriteria decision analysis. Hoboken, NJ: John Wiley & Sons.

[17] Nedjah N, de Macedo Mourelle L. 2005 Evolutionary multi-objective optimisation: a review. In *Real-world multi-objective system engineering* (eds N Nedjah, L de Macedo Mourelle), pp. 3–27. New York, NY: Nova Science.

[18] Malczewski J. 2000 On the use of weighted linear combination method in GIS: common and best practice approaches. Trans. GIS **4**, 5-22. (10.1111/1467-9671.00035)

[19] Mateo J. 2012 Weighted sum method and weighted product method. In *Multi criteria analysis in the renewable energy industry*, pp. 19–22. Berlin, Germany: Springer Science & Business Media.

[20] Chakhar S, Mousseau V. 2008 Spatial multicriteria decision making. Encycl. GIS **10**, 978. (10.1007/978-3-319-17885-1_839)

[21] León R, Shoemaker A, Kacker R. 1987 Performance measures independent of adjustment: an explanation and extension of Taguchi’s signal-to-noise ratios. Technometrics **29**, 253-265. (10.2307/1269331)

[22] Wilding T, Stoeck T, Morrissey B, Carvalho S, Coulson M. 2023 Maximising signal-to-noise ratios in environmental DNA-based monitoring. Sci. Total Environ. **858**, 159735. (10.1016/j.scitotenv.2022.159735) 36349630

[23] Rajakaruna H, Drake D, Bailey S. 2016 Optimizing performance of nonparametric species richness estimators under constrained sampling. Ecol. Evol. **6**, 7311-7322. (10.1002/ece3.2463) 28725399 PMC5513256

[24] Weiss D et al. 2018 A global map of travel time to cities to assess inequalities in accessibility in 2015. Nature **553**, 333-336. (10.1038/nature25181) 29320477

[25] Davidson G, Chua T, Cook A, Speldewinde P, Weinstein P. 2019 The role of ecological linkage mechanisms in *Plasmodium knowlesi* transmission and spread. EcoHealth **16**, 594-610. (10.1007/s10393-019-01395-6) 30675676

[26] Potapov P et al. 2008 Mapping the world’s intact forest landscapes by remote sensing. Ecol. Soc. **13**, 16. (10.5751/ES-02670-130251)

[27] Goldberg DE. 1989 Genetic algorithms in search, optimization and machine learning. Reading, MA: Addison-Wesley.

[28] Chao C, Thompson S. 2001 Optimal adaptive selection of sampling sites. Environmetrics **12**, 517-538. (10.1002/env.477)

[29] World Health Organization. 2020 *World malaria report 2020*. See https://www.who.int/publications/i/item/9789240015791.

[30] Herdiana H et al. 2018 Two clusters of *Plasmodium knowlesi* cases in a malaria elimination area, Sabang Municipality, Aceh, Indonesia. Malar. J. **17**, 1-10. (10.1186/s12936-018-2334-1) 29720188 PMC5932826

[31] Moyes C et al. 2016 Predicting the geographical distributions of the macaque hosts and mosquito vectors of *Plasmodium knowlesi* malaria in forested and non-forested areas. Parasit. Vectors **9**, 242. (10.1186/s13071-016-1527-0) 27125995 PMC4850754

[32] Coutrier F et al. 2018 Laboratory challenges of *Plasmodium* species identification in Aceh Province, Indonesia, a malaria elimination setting with newly discovered *P. knowlesi*. PLoS Negl. Trop. Dis. **12**, e0006924. (10.1371/journal.pntd.0006924) 30500828 PMC6291163

[33] Cooper D et al. 2020 *Plasmodium knowlesi* malaria in Sabah, Malaysia, 2015–2017: ongoing increase in incidence despite near-elimination of the human-only *Plasmodium* species. Clin. Infect. Dis. **12**, e0006924. (10.1371/journal.pntd.0006924) PMC7768742 30889244

[34] Fornace K et al. 2019 Environmental risk factors and exposure to the zoonotic malaria parasite *Plasmodium knowlesi* across northern Sabah, Malaysia: a population-based cross-sectional survey. Lancet Planet. Health **3**, e179-e186. (10.1111/j.1472-4642.2007.00341.x) 31029229 PMC6484808

[35] Grigg M et al. 2017 Individual-level factors associated with the risk of acquiring human *Plasmodium knowlesi* malaria in Malaysia: a case-control study. Lancet Planet. Health **1**, e97-e104. (10.1016/S2542-5196(17)30031-1) 28758162 PMC5531251

[36] Pramasivan S et al. 2021 Spatial distribution of *Plasmodium knowlesi* cases and their vectors in Johor, Malaysia: in light of human malaria elimination. Malar. J. **20**, 1-12. (10.1186/s12936-021-03963-0) 34715864 PMC8555301

[37] Hod R, Mokhtar S, Muharam F, Shamsudin U, Hisham Hashim J. 2021 Developing a predictive model for *Plasmodium knowlesi*–susceptible areas in Malaysia using geospatial data and artificial neural networks. Asia Pac. J. Public Health **34**, 322. (10.1177/10105395211048620) 34569889

[38] Chang W, Cheng J, Allaire J, Xie Y, McPherson J. 2015 Package ‘shiny’. See http://citeseerx.ist.psu.edu/viewdoc/download.

[39] Harrison L. 2023 lu-harr/pk_multicriteria_framework: release to be submitted with paper. Zenodo. (10.5281/zenodo.10247166)

[40] Harrison LE, Flegg JA, Tobin R, Lubis IND, Noviyanti R, Grigg MJ, Shearer FM, Price DJ. 2024 A multi-criteria framework for disease surveillance site selection: case study for *Plasmodium knowlesi* malaria in Indonesia. Figshare. (10.6084/m9.figshare.c.6996704) PMC10776229 38204787

